# Maintaining a gluten-free diet is associated with quality of life in youths with type 1 diabetes and celiac disease

**DOI:** 10.1007/s00592-024-02281-6

**Published:** 2024-04-13

**Authors:** Roberto Franceschi, Riccardo Pertile, Marco Marigliano, Enza Mozzillo, Claudio Maffeis, Francesca Di Candia, Ludovica Fedi, Dario Iafusco, Angela Zanfardino, Stefano Passanisi, Fortunato Lombardo, Maurizio Delvecchio, Gaia Caldarelli, Alda Troncone

**Affiliations:** 1 Department of Pediatrics, S. Chiara Hospital of Trento, APSS, Trentino-Alto Adige, Trento, Italy; 2 Clinical and Evaluative Epidemiology Unit, Department of Governance, APSS, Trento, Italy; 3 Department of Surgery, Dentistry, Pediatrics, and Gynecology, Section of Pediatric Diabetes and Metabolism, University and Azienda Ospedaliera, Universitaria Integrata of Verona, Piazzale Aristide Stefani 1, 37126 Verona, Italy; 4 Department of Translational Medical Science, Section of Pediatrics, Università Degli Studi Di Napoli Federico II, Naples, Italy; 5 Department of Woman, Child, and General, and Specialistic Surgery, Regional Center of Pediatric Diabetes, the University of Campania “L. Vanvitelli”, Naples, Italy; 6 Department of Human Pathology of Adulthood and Childhood G. Barresi, University of Messina, Messina, Italy; 7 Department of Biotechnological and Applied Clinical Sciences (DISCAB), University of L’Aquila, Via Vetorio, Coppito 2, L’Aquila, Italy; 8 Department of Psychology, The University of Campania “L. Vanvitelli”, Caserta, Italy

**Keywords:** QoL, Celiac disease, Diabetes, Young people, Diet

## Abstract

**Aim:**

Conflicting findings have been reported on whether in youths, the double diagnosis of type 1 diabetes (T1D) and celiac disease (CD) substantially impacts quality of life QoL, compared to subjects with T1D only.

**Methods:**

In this study, 86 youths with double diagnosis and their parents were compared to 167 subjects with T1D only. QoL was assessed through the KINDL questionnaire. Anti-tissue transglutaminase antibodies and dietary interviews evaluated the degree of maintaining a gluten-free diet (GFD).

**Results:**

We found that having CD in addition to T1D has little effect on overall QoL. However, analysis of the degree of maintaining GFD revealed significantly lower total QoL scores in groups with T1D + CD not strictly maintaining GFD compared to T1D only (*p* = 0.0014). The multivariable linear regression model confirmed the importance of maintaining GFD on QoL in subjects (*p* = 0.0066) and parents (*p* = 0.023).

**Conclusion:**

The coexistence of T1D and CD and the adoption of a GFD resulted in poor QoL levels, as in youth as in their parents, when difficulties implementing the GFD are present. Psychological support should consider the importance of maintaining GFD not only to prevent potential complications in the future but also to improve actual QoL in different subdomains.

**Supplementary Information:**

The online version contains supplementary material available at 10.1007/s00592-024-02281-6.

## Introduction

Subjects with type 1 diabetes (T1D) are routinely screened for celiac disease (CD), and the importance of diagnosis and treating CD lies with the potential complications: osteoporosis, iron-deficiency anemia, lymphoma, small-bowel cancer, and frequent episodes of hypoglycemia [1]. Both CD and T1D are chronic illnesses found to be related to higher vulnerability to psychological problems, including anxiety and depression [2, 3], and have an impact on quality of life (QoL) [4, 5].

Despite the higher prevalence of CD in children with T1D (from 1.9 to 6.9%) compared with the general population (0.5%) [6, 7], the repercussions of dietary and lifestyle changes imposed by both diseases on QoL have been poorly evaluated. In a recent systematic review, we analyzed data on QoL in pediatric subjects with CD in addition to T1D and found a paucity of data [8]. In three studies, parental and child reports showed that the coexistence of T1D and CD does not substantially impact QoL, and it appears that having the CD in addition to T1D has little effect on QoL [8–11]. These data were collected on small cohorts of 14 to 35 children with T1D and CD compared to pairs with T1D alone [9–11]. In addition, in the studies mentioned above, QoL was mainly assessed through instruments that primarily focused attention on areas of diabetes-specific QoL, overlooking dimensions strictly related to general subjective well-being such as self-esteem and family functioning [9, 10]. In adults, the coexistence of T1D and CD was associated with lower generic and diabetes-specific QoL in one study [12] but not in another [13] that observed that individuals with both diseases tended to achieve higher QoL scores, which means better general health when compared with T1D only, CD only or control subjects.

Given these conflicting findings, in this study, we aimed to obtain more insights into the association between the coexistence of T1D and CD and QoL to understand if subjects with dual conditions require additional care. Two were the main objectives of this study:

1. Analyze differences in QoL between subjects with double diagnosis (T1D and CD) vs. subjects with T1D;
2. Identify the main predictors of QoL in T1D and CD samples.

Moreover, in examining QoL, we also considered maintaining a gluten-free diet (GFD) degree. There is mixed evidence suggesting that youth with T1D and CD who are low maintaining GFD reported lower well-being and diabetes-specific QoL [10], along with worse glycemic control in terms of HbA1c [10, 14], and that QoL does not differ between youth who maintain GFD (GFD +) compared to who do not maintain (GFD −) [9].

## Methods

### Participants and procedure

We conducted an observational study including children and adolescents aged 8–18 years with T1D and CD and their parents attending six pediatric diabetes clinics for three-monthly scheduled visits. All centers belonged to the Italian Society for Pediatric Endocrinology and Diabetes (Bari, Messina, Napoli Federico II, Napoli G. Stoppoloni, Trento, and Verona). The inclusion criteria, as previously reported [14, 15], were: (1) age 8–18 years at the time of recruitment, with a diagnosis of T1D and diabetes duration > 1 year for both T1D and CD group, and T1D only group; (2) to be on the same treatment modality from at least 2 months; (3) diagnosis of CD demonstrated by performing small bowel biopsy or by a biopsy sparing approach according to ESPGHAN guidelines in effect at the time of diagnosis of CD [14, 15]; (4) to be on a GFD for at least 12 months. Exclusion criteria were: (1) fail to meet the inclusion criteria; (2) the presence of complications related to diabetes (peripheral nerve abnormality, retinopathy, renal disease); (3) presence of other autoimmune diseases.

The comparator group (subjects with T1D only) was enrolled consecutively with the proportion 2:1, and its characteristics were previously reported [14]. Data were collected from 01/06/2023 to 30/08/2023.

The Clinical Research Ethics Committee (128/2023) of the coordinating center of Naples reviewed and approved the study, which the Helsinki Declaration conducted. Written informed assents and consents were obtained by minors aged ≥ 12 and all parents before study entry.

### Measures

An examination of participants' clinical records was systematically conducted to collect diabetes-related data (age at diabetes onset, HbA1c at the enrollment, insulin treatment modality, total daily dose, time in range 3.9–10 mmol/L—TIR), BMI z-score at the enrollment, and CD related data (age and HbA1c at CD onset, presence of symptoms at the diagnosis, anti-tissue transglutaminase antibodies (tTG) levels, small bowel biopsy histology).

The GFD maintaining degree in participants with CD and T1D was assessed through tTG titer and a dietary interview conducted by expert dietitians from the tertiary-level pediatric diabetes centers participating in the study [14]. During the dietician’s interview, maintaining the Mediterranean diet was also assessed using the validated Italian version of the Mediterranean Diet Quality Index (KIDMED) score [14].

QoL was assessed through the KINDL questionnaire in T1D and CD subjects and T1D only [16] and was completed by subjects (at least 8, but not yet 18 years old) and parents. The psychometric testing of the KINDL indicates adequate to good reliability and convergent and discriminant validity of this inventory [17], and the KINDL was proved efficient in the studies on HRQoL in children with T1DM [18].

It comprises 31 questions spread across seven scales. Each scale corresponds to a specific dimension of health-related QoL: physical well-being (items 1–4), emotional well-being (items 5–8), self-esteem (items 9–12), family (items 13–16), friends (items 17–20), daily routine (school, items 11–24). Seven more items (from 25 to 31) correspond to the disease module. The score attributed to each answer goes from 1 (never) to 5 (always) for questions with a positive direction and from 5 (never) to 1 (always) for the negative ones. Scores can be expressed either by addition or by the mean. Overall, the KINDL score was calculated after the “reverse scoring” of the negative items, such that higher scores indicate greater QoL.

### Outcomes

KINDL total score for each dimension was calculated and reported in the results section. Demographic and clinical data and glucose control parameters have been reported elsewhere [14].

### Statistical analysis

Analyses were conducted using SAS v9.1.4. (SAS Institute Inc., Cary, NC). The sample size was calculated in collaboration with biostatisticians during the planning of a previous study on glucose metrics in subjects with T1D + CD, compared with T1D only [14], considering TIR as the main outcome of the study, and the number of 18 subjects for each group was suggested. Categorical variables are presented as observed frequencies and percentages, while quantitative variables are presented as mean ± SD and median. The Kolmogorov–Smirnov test was used to verify the normality of distributions.

Student t test for two independent samples was used to compare quantitative measures when normally distributed, otherwise the nonparametric Kruskal–Wallis test was used. Chi-squared test was performed to evaluate associations between categorical variables. Wilcoxon–Mann–Whitney test was used to compare different groups of subjects or parents. Wilcoxon signed rank sum test was conducted to compare matched numerical variables in subjects and parents.

Correlations between KINDL scores and sociodemographic and diabetes-related variables (i.e., illness duration, HbA1c, TIR, time above range > 13.9 mmol/L- TAR) in subjects were calculated using the Spearman correlation coefficient. Multivariable linear regression analysis was chosen to identify independent predictors of KINDL score (subjects and parents). The significance level was set to a p-value ≤ 0.05. Data processing has been entrusted to the Governance Department of the Clinical and Evaluation Epidemiology Service of Azienda Provinciale Per i Servizi Sanitari del Trentino (APSS).

## Results

Of the 108 subjects with T1D and CD invited to participate, 22 refused (most of them were in a hurry or their parents said they were not interested); the study samples consisted of 86 subjects with T1D and CD and their parents and 167 matched control subjects with T1D. Relevant population characteristics for this study are reported in Table 1, and details are written in a previous manuscript [14]. No statistically significant differences were found between the two groups except for age at diabetes onset (p = 0.003) and diabetes duration (p = 0.006). They presented similar growth parameters, total daily insulin dose, HbA1c, and TIR (all p > 0.05). Sixty-one (71%) youths were strictly maintaining GFD (GFD +), and 25 (29%) were not wholly (GFD-), as assessed by the dietician interview, which was more sensitive than tTG titer to detect lapses in the diet. GFD- subjects were older at diabetes onset (p = 0.009), and consequently, they presented shorter diabetes duration at the time of enrollment (p = 0.013). No differences were found between GFD + and GFD- groups in terms of gender distribution (p = 0.428), age at study enrollment (p = 0.658), symptoms at CD diagnosis (p = 0.977), insulin treatment (p = 0.127), BMI (p = 0.571), HbA1c levels (p = 0.082), daily insulin dose (p = 0.645) and TIR (p = 0.130).

**Table 1:** Descriptive statistics and glucose metrics of people with type 1 diabetes and celiac disease compared to ones with type 1 diabetes only, at the study enrollment

	T1D and CD	T1D only	p-value T1 and CD vs. T1D	GFD-	GFD +	p-value GFD- vs. GFD +
Sample size	86	167		25	61	
Female n (%)	47 (55%)	76 (45.5%)	0.168^a^	12 (48%)	35 (57%)	0.428^a^
Age at study enrollment (years)	13.8 ± 2.6 (14.2)	13.6 ± 2.9 (13.7)	0.750^b^	13.6 ± 3.0 (14.9)	13.9 ± 2.5 (14.0)	0.658^b^
Age at diabetes onset (years)	6.2 ± 4.0 (5.9)	7.7 ± 3.6 (7.4)	**0.003**^**c**^	8.0 ± 4.2 (7.1)	5.5 ± 3.8 (4.6)	**0.009**^**c**^
Symptoms of CD at the diagnosis (%)	32%	n.a		32%	31%	0.977^a^
Diabetes duration (years)	7.6 ± 4.4 (7.8)	5.95 ± 3.6 (5.6)	**0.006**^b^	5.6 ± 4.6 (3.4)	8.4 ± 4.1 (8.5)	**0.013**^b^
Insulin treatment *n* (%)						
MDI	47 (54%)	86 (52%)	0.960^a^	18 (72%)	29 (47%)	0.127^a^
SAP	16 (19%)	32 (19%)		5 (20%)	14 (23%)	
HCL	6 (7%)	14 (8%)		0 (0%)	3 (5%)	
AHCL	17 (20%)	35 (21%)		2 (8%)	15 (25%)	
BMI z-score	0.3 ± 1.0 (0.3)	0.3 ± 1.0 (0.3)	0.725^**c**^	0.2 ± 1.1 (0.2)	0.4 ± 0.9 (0.4)	0.571^c^
% HbA1c [mean ± SD (Median)]	7.2 ± 1.2 (7.1)	7.03 ± 0.8 (7.0)	0.417^b^	7.5 ± 1.0 (7.2)	7.1 ± 1.2 (7.0)	0.082^b^
HbA1c (mmol/mol)	55 ± 10 (54)	53 ± 15 (53)		58 ± 13 (55)	54 ± 10 (53)	
Total daily insulin dose (IU/Kg) [mean ± SD (Median)]	0.8 ± 0.2 (0.8)	0.7 ± 0.3 (0.7)	0.093^b^	0.8 ± 0.3 (0.8)	0.8 ± 0.3 (0.8)	0.645^c^
% of time in range (3.9–10 mmol/L) (%TIR)	60.6 ± 19.6 (61.0)	63.8 ± 18.3 (68.0)	0.155	55.3 ± 19.7 (53.0)	62.6 ± 19.3 (63.5)	0.130^c^

Data are reported as [mean ± SD (Median)]. MDI: multiple daily injections, SAP: sensor-augmented pump, HCL: hybrid closed loop, AHCL: advanced hybrid closed loop. BMI: body mass index. HbA1c: glycosylated hemoglobin. TIR: time in range. GFD-: not strictly maintaining gluten-free diet, GFD + : strictly maintaining

^a^Chi squared test, ^b^Nonparametric Kruskal–Wallis’ test, ^c^Student’s *t* test with equal variances between groups

GFD- subjects, compared to T1D only reported higher HbA1c (7.5 ± 1.0% vs 7.03 ± 0.8%, p = 0.039) and lower TIR (55.3 ± 19.7% vs 63.8 ± 18.3%, p = 0.039), higher TAR mmol/L (18.3 ± 16.1% vs 11.3 ± 12.1%, p = 0.046) and higher mean glucose (9.9 ± 2.4 vs 9.0 ± 1.5 mmol/L, p = 0.048). Other diabetes-related variables reported in Table 1 were not significantly different between the two groups [14].

### QoL in individuals with T1D and CD and individuals with T1D only (according to individuals and parents’ report)

Children adolescents with T1D and CD and their parents reported more than neutral KINDL mean total scores (range 1 to 5): 3.97 ± 0.54 and 3.88 ± 0.64, respectively (Table 2). In this group, no differences were found between parents and subjects with T1D report in KINDL total score (p = 0.149) and all QoL dimensions considered, regardless of maintaining GFD (p > 0.05) (except for GFD + subjects disease module scores, p = 0.00014).

**Table 2:** KINDL scores in subjects with T1D and CD and their paired parents (*N* = 86). Data are reported as mean ± SD (median). GFD-: not strictly maintaining GFD, GFD + : strictly maintaining

Scores	T1D and CD subjects	T1D and CD parents	*p*-value T1D and CD subjects vs. parents	T1D only subjects	*p*-value Subjects T1D and CD vs. subjectsT1D only	T1D only parents	*p*-value Parents T1D and CD vs. parents T1D only
Overall KINDL							
Total (*N* = 86)	3.97 ± 0.54 (4.14)	3.88 ± 0.64 (4.05)	0.149^#^	4.03 ± 0.41 (4.07)	0.312*	4.04 ± 0.41 (4.12)	0.132*
GFD- (*N* = 25)	3.63 ± 0.68 (3.67)	3.49 ± 0.75 (3.52)	0.298	4.03 ± 0.41 (4.07)	0.085	4.04 ± 0.41 (4.12)	**0.0014**
GFD + (*N* = 36)	4.10 ± 0.40 (4.16)	4.06 ± 0.48 (4.13)	0.430	4.03 ± 0.41 (4.07)	0.150	4.04 ± 0.41 (4.12)	0.342
Physical well-being							
GFD-	3.58 ± 0.93 (4.00)	3.48 ± 0.99 (3.50)	0.607	4.15 ± 0.64 (4.25)	**0.024**	4.14 ± 0.62 (4.25)	**0.0045**
GFD +	4.11 ± 0.59 (4.25)	44.01 ± 0.62 (4.00)	0.185	4.15 ± 0.64 (4.25)	0.432	4.14 ± 0.62 (4.25)	0.085
Emotional well-being							
GFD-	3.50 ± 0.87 (3.50**)**	3.00 ± 1.46 (3.38)	0.461	4.05 ± 0.57 (4.25)	**0.036**	4.07 ± 0.58 (4.25)	**0.0032**
GFD +	4.10 ± 0.48 (4.00)	3.70 ± 1.16 (4.00)	0.345	4.05 ± 0.57 (4.25)	0.318	4.07 ± 0.58 (4.25)	0.122
Self-esteem							
GFD-	3.55 ± 1.06 (3.88)	3.52 ± 0.98 (3.63)	0.654	3.81 ± 0.71 (3.75)	0.360	3.84 ± 0.71 (4.00)	0.118
GFD +	3.99 ± 0.68 (4.00)	3.97 ± 0.64 (4.00)	0.953	3.81 ± 0.71 (3.75)	0.089	3.84 ± 0.71 (4.00)	0.161
Family							
GFD-	3.80 ± 1.01 (4.13)	3.70 ± 0.51 (3.75)	0.302	3.93 ± 0.64 (4.00)	0.247	3.93 ± 0.63 (4.00)	**0.045**
GFD +	4.33 ± 0.56 (4.50)	4.23 ± 0.51 (4.25)	0.163	3.93 ± 0.64 (4.00)	** < 0.0001**	3.93 ± 0.63 (4.00)	**0.0031**
Friends							
GFD-	4.09 ± 0.77 (4.00)	3.86 ± 0.66 (4.00)	0.424	4.18 ± 0.66 (4.25)	0.309	4.20 ± 0.65 (4.25)	**0.016**
GFD +	4.31 ± 0.55 (4.50)	4.24 ± 0.65 (4.50)	0.442	4.18 ± 0.66 (4.25)	0.223	4.20 ± 0.65 (4.25)	0.360
Daily routine (School)							
GFD-	3.31 ± 0.76 (3.25)	3.33 ± 1.04 (3.38)	0.607	3.81 ± 0.72 (3.75)	**0.009**	3.83 ± 0.74 (3.75)	**0.036**
GFD +	3.74 ± 0.73 (3.88)	3.86 ± 0.79 (3.75)	0.608	3.81 ± 0.72 (3.75)	0.444	3.83 ± 0.74 (3.75)	0.375
Disease module							
GFD-	3.55 ± 0.98 (3.42)	3.51 ± 1.02 (3.67)	0.791	4.28 ± 0.53 (4.33)	**0.002**	4.30 ± 0.51 (4.33)	**0.001**
GFD +	4.11 ± 0.81 (4.42)	4.39 ± 0.57 (4.58)	**0.0014**	4.28 ± 0.53 (4.33)	0.310	4.30 ± 0.51 (4.33)	0.087

^*^Wilcoxon–Mann–Whitney test # Wilcoxon signed rank sum test

There was no difference in KINDL total score between subjects with T1D and the ones with T1D and CD (p = 0.312), as well as between scores of parents of children with T1D and parents of children with T1D and CD (p = 0.132) (Table 2).

Analysis according to the GFD maintaining degree showed lower total QoL scores according to parent’s report of subjects with T1D and CD in the GFD- subgroup, compared to scores obtained by parents of subjects with T1D only (p = 0.0014) (Table 2).

Analysis across the different domains showed significantly lower QoL in subjects living with T1D and CD in the GFD- subgroup, compared to T1D-only subjects, in physical, emotional, school, and disease domains (p range: 0.002–0.036). Parents of children with T1D and CD in the GFD- subgroup reported lower QoL scores than parents of children with T1D in the physical (0.0045), emotional (0.0032), school (0.036), friends’ (0.016) and disease (0.001) domains. The GFD + subgroup, according to subjects with T1D (p < 0.0001) and parents report (p = 0.0031), obtained higher family QoL dimension scores than children with T1D (Table 2).

### Predictors of QoL

No significant correlations were found in subjects living with T1D and CD between total QoL scores, age, and diabetes-related variables (Table S1). Only diabetes duration was positively correlated with QoL scores in the “physical well-being” domain; increased TAR was negatively correlated with QoL scores in the “Friends Domain” (Table S1).

In parents, age was negatively correlated with QoL total score (Table S2), TIR was positively correlated with QoL scores in the “physical well-being” domain, while HbA1c and TAR were negatively correlated. HbA1c was negatively correlated with QoL scores in the “daily routine (school)” domain (Table S2).

Multivariable linear regression analysis in subjects with T1D and CD and their parents was performed, using KINDL total score as the outcome variable and as predictors the following variables: age, gender, disease-related variables (illness duration, TIR), insulin treatment modality (MDI vs. others) and maintaining GFD (Table 3). Keeping GFD was significantly and positively associated with KINDL total score in subjects with T1D and CD (p = 0.0066) and parents (p = 0.023) (Table 3); in the last ones, TIR was also associated with KINDL total score (p = 0.0199). No significant correlation was found between other variables and QoL. Multivariable linear regression analysis comparing GFD- and T1D only, subjects with T1D and their parents was performed using the KINDL score as the outcome variable. HbA1c was unrelated to KINDL total score, and GFD- still reported lower scores after excluding the HbA1c effect (subjects with T1D p = 0.0168, parents < 0.0001, data not shown).

**Table 3:** Multivariable linear regression analysis in subjects living with T1D and CD and parents reports independent predictors of KINDL scores. T1D: type 1 diabetes, CD: celiac disease, MDI: multiple daily injections, TIR: time in range, GFD: gluten-free diet

	Subjects		Parents	
	Estimate	*p*-value	Estimate	*p*-value
Gender	0.188	0.182	−0.053	0.763
Age at enrollment	−0.037	0.211	0.004	0.946
T1D duration	0.037	0.131	0.054	0.06
CD duration	−0.018	0.537	−0.057	0.09
MDI vs other treatment modalities	0.101	0.482	−0.125	0.486
TIR	0.0008	0,843	0.012	**0.0199**
GFD maintaining degree	0.851	**0.0066**	0.886	**0.0237**

## Discussion

The present study aimed to investigate QoL in subjects with T1D and CD and their parents using a QoL measure designed to assess the meaningful dimensions of perceived subjective well-being. In participants with T1D and CD, predictors of QoL were also explored. Existing literature on QoL in T1D and CD individuals compared with other subjects with T1D only provides very little evidence; however, it was collected in small samples, so results from previous studies have been inconclusive as to whether subjects with double diagnosis are more likely to have impaired QoL [9, 10]. Sud et al. reported no significant differences in QoL between subjects with established T1D + CD and those with T1D alone, while their parents reported lower social functioning scores [9]. Pham-Short et al. reported lower diabetes-specific QoL and lower general well-being in subjects GFD- and in their parents, compared to T1D only [10]. O’Neill et al. found no significant differences in QoL in subjects with dual diagnosis, but significant stress for parents was reported [11].

In the present study, T1D and CD, compared to the T1D group, differed very little in sociodemographic and diabetes-related characteristics. Subjects with T1D and CD presented with longer diabetes duration, not detected in previous studies [9, 10]. This is consistent with the possibility of developing new associated autoimmune diseases, such as CD, over time. GFD- subjects, compared to those maintaining GFD, presented shorter diabetes duration at the time of enrollment, and probably the double diagnosis later in life could influence the unwillingness to get used to a GFD. These data were not found in previous studies [9, 10] but did not result as an independent predictor of KINDL scores by the multivariate analysis. Analyzing QoL in T1D and CD group, no significant discrepancies between parents and child reports were observed. At first glance, T1D and CD vs. T1D group comparison showed that having CD in addition to T1D has little effect on overall QoL as in subjects as well as in parents. However, analysis of parent’s reports, according to the degree of maintaining GFD, revealed significantly lower total QoL scores in the group with T1D and CD not strictly maintaining the diet compared to scores obtained by parents of subjects with T1D only.

Such result is in line with previous studies in which the additional diagnosis of CD and starting GFD was found to be a cause of significant stress for parents [11], and whose results indicated that parents of subjects not maintaining GFD reported lower QoL [10]. In contrast, Sud et al. did not find differences in QoL in the T1D and CD group with regard to age at CD diagnosis, CD duration, or on the basis of GFD maintaining degree [9]. However, in interpreting this inconsistency it should be noted that in Sud et al.’s study, the number of youths who were GFD- was very small (n = 6) [9].

Moreover, in the present study analysis across the different domains confirmed lower QoL in both parents and individuals with T1D and CD and GFD- compared to T1D only subjects, revealing lower well-being specifically in physical, emotional, school, and disease domains (p range: 0.002–0.036 in subjects and 0.001–0.045 in parents). Additionally, parents of children with T1D and CD in the GFD- subgroup, but not children, reported also lower QoL in the family (p = 0.045) and friends’ (p = 0.016) domains as compared to parents of children with T1D only. A multivariable linear regression model including variables previously reported in literature as predictors of QoL in T1D and CD population [9, 10], and adding TIR, confirmed the importance of maintaining GFD on QoL both in subjects and parents. In parents but not in subjects, TIR was probably perceived as associated with QoL because they feel more responsibility to achieve good metabolic control to prevent complications, than their children-adolescents can experience.

Considering the lack of substantial differences between those strictly maintaining GFD and who were not (also in terms of symptoms at CD diagnosis, p = 0.977), the lower scores reported in most of the QoL dimensions considered by T1D and CD group who were in the GFD- subgroup, maybe the expression of the general difficulty experienced by subjects and parents, already coping with a chronic disease, to live with a double diagnosis. CD in subjects with T1D is often diagnosed during routine screening, and so subjects generally experience subtle or no gastrointestinal symptoms. Subjects with T1D who receive this additional diagnosis must accept a further restrictive diet without necessarily experiencing physical benefits or improvement in metabolic control. Therefore, the lower QoL that we found in parents and youth who have difficulty implementing the GFD may be owing to greater specific difficulty with the GFD. We speculate that the need to follow a lifelong diet may be probably perceived as a further demanding task to be added to the daily effort to manage T1D which can lead some subjects (and parents) to have a poor degree of maintaining GFD and impairment of subjective well-being. In particular, T1D and CD subjects who were in the GFD- subgroup, were found to experience lower QoL than T1D participants not only in terms of physical well-being (most likely due to poorer physical functioning due to bodily symptoms resulting from poor diet maintenance), but also in terms of emotional, daily/scholastic functioning and disease perception. Our results suggested that heightened dietary restrictions imposed on T1D subjects with CD may result in facing adding difficulties in fully participating in daily and scholastic activities (e.g., due to problems in having gluten-free foods readily available) as well as experiencing further emotional burden and worsened illness perception. It should be considered that during adolescence, participating in daily activities, especially at school with peers, plays a significant role in developing self-identity and maintaining well-being [19]. Therefore, it is reasonable to suppose that the restrictive nature of a GFD, combined with typical developmental issues that adolescents have to face and all tasks resulting from managing a lifelong health condition, may impact a child’s emotional well-being and exacerbate the subjective perception of living in an unhealthy body. According to the present results, this appears to be particularly true for those experiencing difficulties in adhering to a GFD, suggesting a negative and circular relationship between physical, emotional, family, and daily subjective well-being and challenges in maintaining such a demanding dietary regimen.

Moreover, lower well-being also in family and friends’ dimensions reported solely by parents seem to indicate that they perceived CD management as associated with difficulties in family functioning and social situations [10, 20]. It is reasonable to assume that tasks imposed by the double diagnosis (especially those to be carried out at home, like planning meals, having gluten-free foods readily available for their children when they must eat out, etc.) can potentially favor parents adding disease burden which in turn can negative impact on family functioning [20]. Consistent with these suppositions, according to the present findings, both parents and children with T1D and CD who were in the GFD + subgroup perceived QoL for family dimensions better than the T1D-only group. The KINDL measure revealed that their familial context was perceived as better organized, and the meal was well controlled; consequently, subjects felt protected, and this probably generated fewer conflicts compared to subjects with T1D only. Youths who were GFD + perceived lower QoL than their parents in the disease module, probably because they felt having a double disease was an important burden and considered judgment by others as an issue. Similarly, given the increased dietary limitations that individuals with T1D and CD must contend with during social interactions, it seems reasonable for parents to believe that their children may encounter additional challenges when they are with their friends. While gluten-free food options might be easily accessible for children within the confines of their homes, maintaining strictly GFD might restrict their ability to fully engage in activities with their friends like dining out [9].

Potential limitations of this study are: the cross-sectional design, which cannot determine causality; the study was not powered to analyze differences between youths strictly maintaining GFD and not; a small number of not strictly maintaining GFD subjects. Strengths are: the sample size of youths with dual pathology is larger than previously reported; we evaluated maintaining GFD including the dietician interview and the degree of maintaining the Mediterranean diet. Health-related QoL (HRQoL) was assessed through the KINDL questionnaire, in T1 and CD subjects and in T1D only including, along with diabetes-specific aspects, meaning dimension of QoL.

For clinical practice, we emphasize the importance of monitoring the maintenance of GFD in youth with T1D and CD, to identify the GFD– subgroup, that requires additional care: psychological support should consider the importance of maintaining the diet not only to prevent potential complications in the future, but also to improve actual quality of life in different subdomains. We intend to follow strictly up the GFD- subgroup, organizing peer groups that include the intervention of a psychologist and dietician in order to support better compliance with the GFD.

## Conclusions

We found that the coexistence of T1D and CD, and the adoption of a GFD, resulted associated with poor QoL levels, as in youth as in their parents, when difficulties implementing the GFD are present. The lower overall QoL in parents of youth who were in the GFD − subgroup, and in physical, emotional, school, and disease QoL domains in parents and youths, may be owing to perceived greater difficulty with the GFD and to an increased psychological burden related to the CD diagnosis. Parents perceived more problems also in family functioning and children's well-being related to friends, than their children did.

### Supplementary Information

Below is the link to the electronic supplementary material.

Supplementary file1 (DOCX 22 KB)

## Data Availability

Some or all datasets generated during and/or analyzed during the current study are not publicly available but are available from the corresponding author upon reasonable request.

## Abbreviations

QoL: Quality of life
T1D: Type 1 diabetes
CD: Celiac disease
GFD: Gluten-free diet
tTG: Anti-tissue transglutaminase antibodies
TIR: Time in range
TBR: Time below range
TAR: Time above range
