# Supplementary material for: Maintaining a gluten-free diet is associated with quality of life in youths with type 1 diabetes and celiac disease
Source: Acta Diabetol. 2024 Apr 13;61(8):987–95. doi: 10.1007/s00592-024-02281-6 (PMC11329396; doi:10.1007/s00592-024-02281-6)
Supplement: Supplementary file 1 — Supplementary file1 (DOCX 22 KB) [file 592_2024_2281_MOESM1_ESM.docx]

**Table S1:** correlation between KINDL scores in the sub-domains in T1+CD subjects (Spearman correlation coefficient). CD: celiac disease, BMI: body mass index, HbA1c: glycated hemoglobin, TIR: time in range, TAR: time above range.

|  | **KINDL Total score** | **Physical well-being** | **Emotional well-being** | **Self-esteem** | **Family** | **Friends** | **Daily Routine (School)** | **Disease module** |
| --- | --- | --- | --- | --- | --- | --- | --- | --- |
| **Age** | r=-0.1267  p=0.3184 | r=-0.10425  p=0.4123 | r=-0.1227  p=0.3341 | r=-0.17785  p=0.1597 | r=-0.12669  p=0.3185 | r=-0.11347  p=0.372 | r=-0.08656  p=0.4964 | r=-0.06496  p=0.6101 |
| **Diabetes duration** | r=0.18261  p=0.1487 | r= 0.22797 **p=0.07** | r=0.14304  p=0.2595 | r=-0.02433  p=0.8486 | r=0.13359  p=0.2926 | r=0.00957  p=0.9402 | r=0.03483  p=0.7848 | r=0.22915  p=0.0685 |
| **CD duration** | r=0.03785  p=0.7665 | r=0.13602  p=0.2839 | r=-0.0604  0.6354 | r=-0.06766  p=0.5952 | r=0.04277  0.7372 | r=-0.16029  p=0.2058 | r=0.04385  p=0.7308 | r=0.0789  p=0.5355 |
| **BMI Z-score** | r=-0.01292  p=0.9193 | r=0.04385  p=0.7308 | r=-0.05542  0.6636 | r=-0.04072  p=0.7494 | r=0.12273  p=0.334 | r=-0.09168  0.4712 | r=0.00888  0.9445 | r=-0.06035  0.6357 |
| **HbA1c** | r=-0.10398  p=0.4135 | r=-0.11382  p=0.3705 | r=0.08876  p=0.4855 | r=-0.13261  p=0.2962 | r=-0.01396  p=0.9128 | r=-0.26815  p=0.1841 | r=-0.10111  p=0.4266 | r=-0.10164  p=0.4242 |
| **TIR (3.9-10 mmol/L)** | r=0.10801  p=0.3995 | r=0.1185  p=0.3548 | r=-0.08523  p=0.5066 | r=0.14628  p=0.2526 | r=-0.0157  p=0.9028 | r=0.18848  p=0.139 | r=0.13497  p=0.2916 | r=0.04136  p=0.7475 |
| **TAR  (>10 mmol/L)** | r=-0.26611  p=**0.035** | r=-0.1803  p=0.1573 | r=-0.21238  p=0.0947 | r=-0.17916  p=0.16 | r=-0.05719  p=0.6562 | r=-0.37631  p=**0.0024** | r=-0,05898  p=0.6461 | r=-0.15242  p=0.233 |

**Table S2:** correlation between KINDL scores in the sub-domains in T1+CD parents (Spearman correlation coefficient). CD: celiac disease, BMI: body mass index, HbA1c: glycated hemoglobin, TIR: time in range, TAR: time above range.

|  | **KINDL Total score** | **Physical well-being** | **Emotional well-being** | **Self-esteem** | **Family** | **Friends** | **Daily Routine (School)** | **Disease module** |
| --- | --- | --- | --- | --- | --- | --- | --- | --- |
| **Age** | r=-0.25356  **p=0.0432** | r=-0.06509  p=0.6568 | r=-0.15704  p=0.2812 | r=-0.33905  p=0.1134 | r=0.09261  p=0.5268 | r=-0.01285  p=0.9302 | r=-0.0483  p=0.741 | r=-0.0274  p=0.8517 |
| **Diabetes duration** | r=-0.16674  p=0.1879 | r= 0.2053 p=0.157 | r=0.04602  p=0.7535 | r=-0.11354  p=0.4373 | r=0.11064  p=0.4492 | r=0.07847  p=0.592 | r=0.13794  p=0.3446 | r=0.1692  p=0.2452 |
| **CD duration** | r=-0.19657  p=0.1195 | r=0.06653  p=0.6497 | r=-0.10569  p=0.4698 | r=-0.19635  p=0.1763 | r=0.02133  0.8843 | r=-0.07661  p=0.6008 | r=-0.01158  p=0.9371 | r=0.06343  p=0.665 |
| **BMI Z-score** | r=-0.20083  p=0.1116 | r=0.11927  p=0.4144 | r=-0.04499  0.7589 | r=-0.0415  p=0.7771 | r=-0.00021  p=0.9989 | r=-0.02027  p=0.89 | r=-0.05513  0.7068 | r=-0.04295  p=0.7695 |
| **HbA1c** | r=0.086  p=0.4992 | r=-0.06157  p=0.6743 | r=-0.34367  **p=0.0156** | r=-0.10352  p=0.4791 | r=-0.22322  p=0.1231 | r=-0.2717  p=0.059 | r=-0.33551  **p=0.0184** | r=-0.18205  p=0.2106 |
| **TIR (3.9-10 mmol/L)** | r=-0.11794  p=0.3573 | r=0.2586  p=0.0728 | r=0.48638  **p=0.0004** | r=0.11349  p=0.4375 | r=0.26715  p=0.0635 | r=0.19041  p=0.19 | r=0.23208  p=0.1086 | r=0.27115  p=0.0595 |
| **TAR (>10 mmol/L)** | r=0.11456  p=0.3713 | r=-0.20193  p=0.1641 | r=-0.37167  **p=0.0086** | r=-0.06865  p=0.6393 | r=-0.21379  p=0.1402 | r=-0.19113  p=0.1883 | r=-0,17874  p=0.2191 | r=-0.26914  p=0.0615 |
